# First person – Laura Tamberg

**DOI:** 10.1242/dmm.046532

**Published:** 2020-07-30

## Abstract

First Person is a series of interviews with the first authors of a selection of papers published in Disease Models & Mechanisms, helping early-career researchers promote themselves alongside their papers. Laura Tamberg is first author on ‘Daughterless, the *Drosophila* orthologue of TCF4, is required for associative learning and maintenance of the synaptic proteome’, published in DMM. Laura is a PhD student in the lab of Tõnis Timmusk at the Tallinn University of Technology, Tallinn, Estonia. Her research involves investigating the use of *Drosophila melanogaster* as a model system to understand the molecular mechanisms underlying transcription factor TCF4-related neuronal diseases.


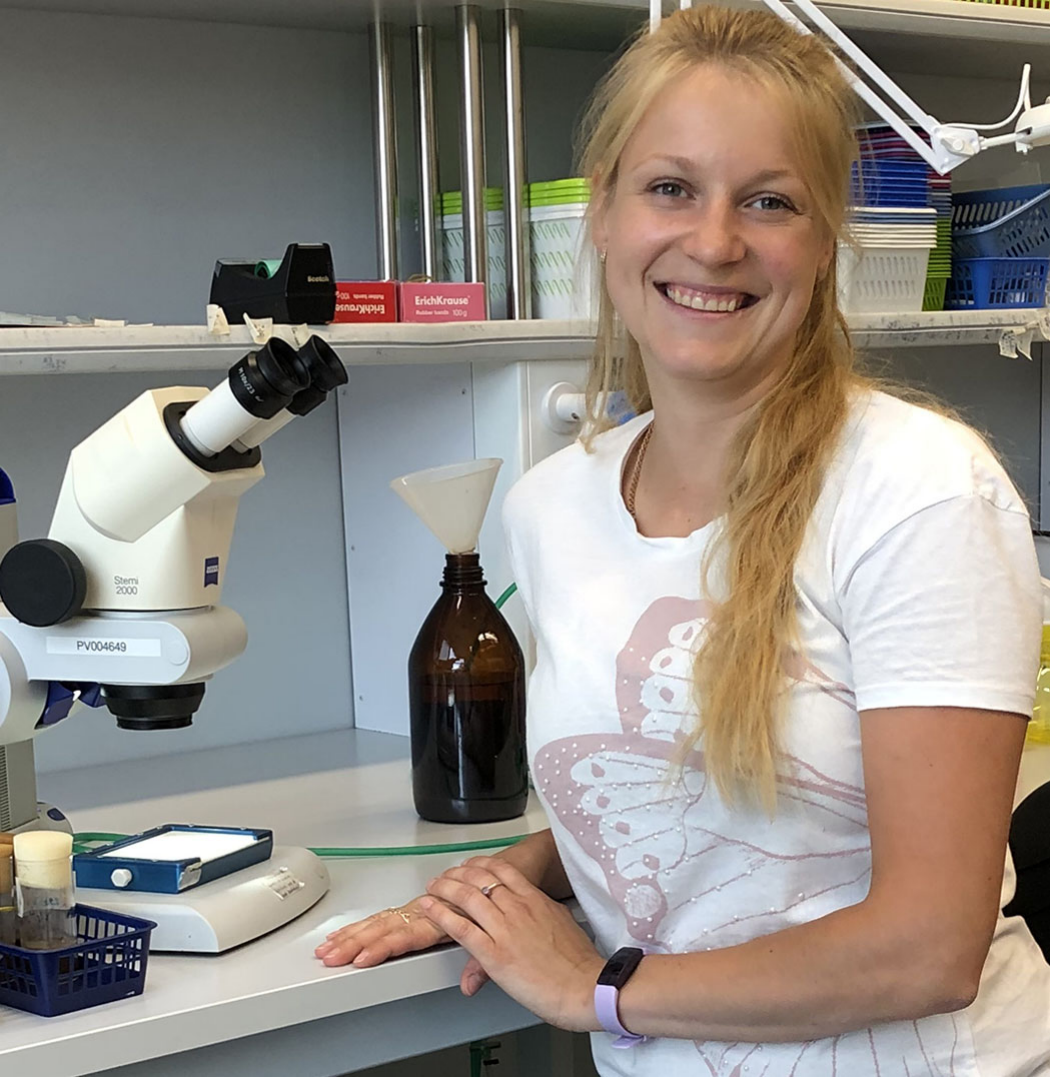


**Laura Tamberg**

**How would you explain the main findings of your paper to non-scientific family and friends?**

*Drosophila melanogaster*, commonly known as the fruit fly, is widely used as a model system for human diseases as at least 70% of human disease-related genes have corresponding genes in the fly. We used the fruit fly to advance knowledge about the transcription factor TCF4. Mutations in the *TCF4* gene cause Pitt–Hopkins syndrome and mild intellectual disability. Also, variations in the human *TCF4* gene have been associated with schizophrenia and bipolar disease. We found that Daughterless, the fruit fly equivalent of TCF4, affects learning and memory in *Drosophila* larvae and locomotion in adult flies. These phenotypes can be used for modelling some aspects of Pitt–Hopkins syndrome, which is characterized by intellectual disability, locomotor difficulties and more. Learning and locomotor deficits in our fruit fly models could be caused by dysregulation of the synaptic proteome, as we found two synaptic proteins to be downregulated. Also, we tested two substances, resveratrol and suberanilohydroxamic acid (SAHA), the last of which is known to improve memory in the mouse model of Pitt–Hopkins syndrome. First, we detected strong enhancement of both Daughterless and TCF4 protein activity by these substances in a cell-based assay. Second, both resveratrol and SAHA had a moderate positive effect on learning and memory in fruit fly larvae, and SAHA improved the locomotion of adult flies in our model.

“Our fruit fly models can be used to further screen therapeutics to improve some aspects of Pitt–Hopkins syndrome.”

**What are the potential implications of these results for your field of research?**

Our fruit fly models can be used to further screen therapeutics to improve some aspects of Pitt–Hopkins syndrome. Also, these models can be used to give novel insights into the molecular mechanisms of TCF4-associated diseases.

**What are the main advantages and drawbacks of the model system you have used as it relates to the disease you are investigating?**

*Drosophila* has a short lifecycle, so it is suitable for screening for potential therapeutics for Pitt–Hopkins syndrome. Our first model, in which we used the larval learning paradigm, is quite time consuming but measuring locomotion in the second model is quick and easy, and provides a powerful tool. A drawback of using fruit flies to model Pitt–Hopkins syndrome is that in humans haploinsufficiency of TCF4 causes severe symptoms associated with the syndrome but in the fly haploinsufficiency of Daughterless has no phenotype so we silenced the gene. Nevertheless, the observed phenotypes were similar to several Pitt–Hopkins syndrome symptoms.

**What has surprised you the most while conducting your research?**

Using the appetitive associative learning paradigm with fruit fly larvae was surprising. This is a Pavlovian conditioning test in which larvae learn an odour that is presented with a sugar reward and they then move towards the odour in the test situation. Surprisingly, larvae with lowered levels of Daughterless in the brain had impaired learning ability, which is in a way similar to Pitt–Hopkins patients having intellectual disability.

**Figure:**
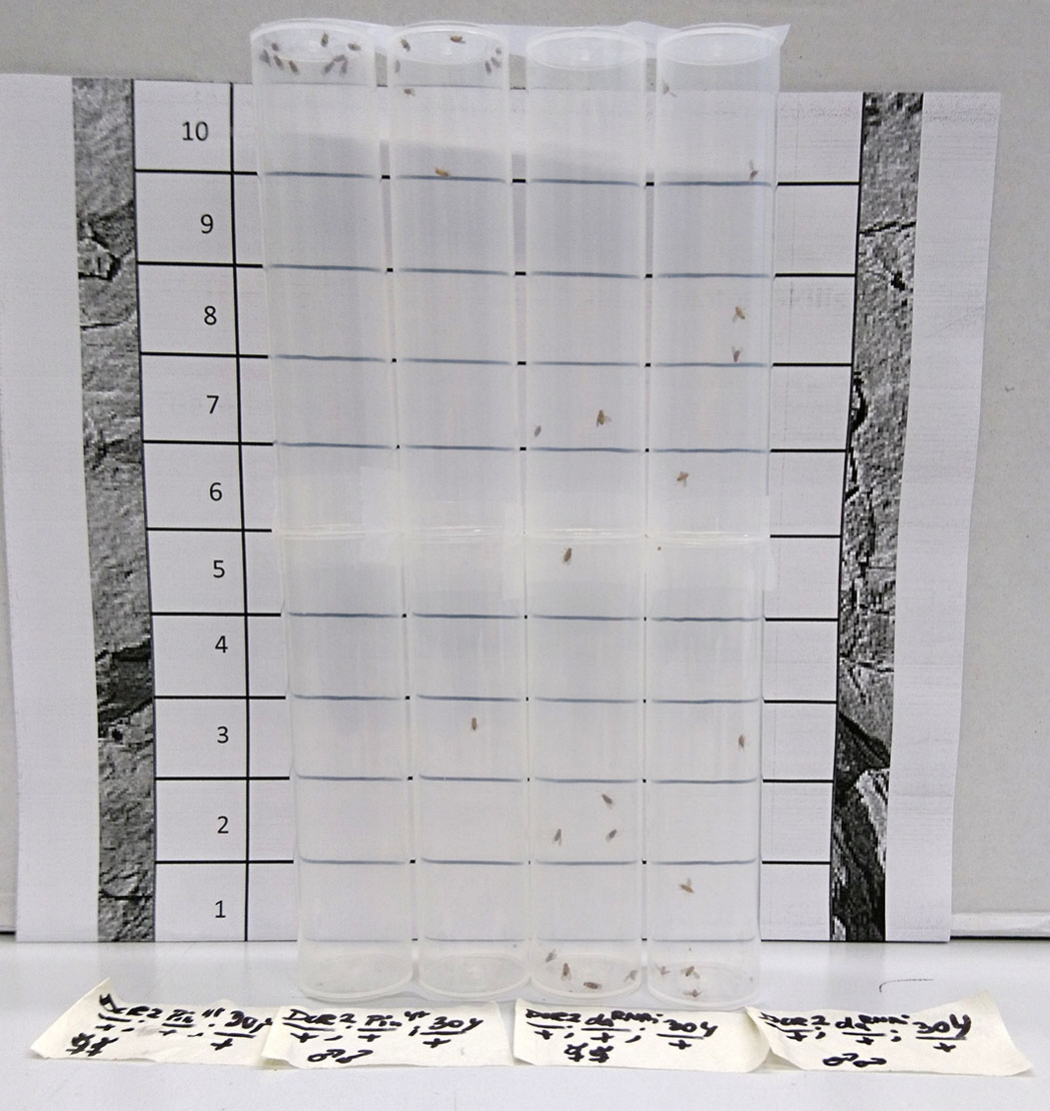
**Negative geotaxis of *Drosophila* is impaired when *daughterless* is silenced in the brain.** Wild-type fruit flies move upwards, which is a behaviour called negative geotaxis. This behaviour is affected by Daughterless, a transcription factor that is homologous to human TCF4.

**What changes do you think could improve the professional lives of early-career scientists?**

I think early-career scientists could be encouraged to network worldwide even more and share experiences from different countries and labs.

**What's next for you?**

This is my second article about Pitt–Hopkins syndrome in *Drosophila*. Here in Estonia, a PhD student needs three papers for their doctoral thesis. My third article will be about Daughterless target genes as this will give insights about possible TCF4 targets as well. I have already conducted chromatin immunoprecipitation (ChIP) sequencing analysis of fruit fly heads. Next, I will carry out RNA-seq analysis of *Drosophila* brains in which *daughterless* has been silenced or overexpressed and then compare these to controls. Using these two data sets, ChIP-seq and RNA-seq, I will be able to determine the direct target genes of Daughterless. After publication of my third article, I can start writing my thesis.
